# Sudden cardiac death risk in hypertrophic cardiomyopathy: comparison between echocardiography and magnetic resonance imaging

**DOI:** 10.1038/s41598-021-86532-4

**Published:** 2021-03-30

**Authors:** Mateusz Śpiewak, Mariusz Kłopotowski, Ewa Kowalik, Agata Kubik, Natalia Ojrzyńska-Witek, Joanna Petryka-Mazurkiewicz, Ewa Michalak, Łukasz Mazurkiewicz, Monika Gawor, Katarzyna Kożuch, Barbara Miłosz-Wieczorek, Jacek Grzybowski, Zofia Bilińska, Adam Witkowski, Anna Klisiewicz, Magdalena Marczak

**Affiliations:** 1 Magnetic Resonance Unit, Department of Radiology, National Institute of Cardiology, ul. Alpejska 42, 04-628 Warsaw, Poland; 2 Department of Cardiology and Interventional Angiology, National Institute of Cardiology, Warsaw, Poland; 3 Department of Congenital Heart Diseases, National Institute of Cardiology, Warsaw, Poland; 4 Department of Cardiomyopathies, National Institute of Cardiology, Warsaw, Poland; 5 Department of Coronary Artery Disease and Structural Heart Diseases, National Institute of Cardiology, Warsaw, Poland; 6 Unit for Screening Studies in Inherited Cardiovascular Diseases, National Institute of Cardiology, Warsaw, Poland

**Keywords:** Cardiology, Cardiac hypertrophy

## Abstract

In hypertrophic cardiomyopathy (HCM) patients, left ventricular (LV) maximal wall thickness (MWT) is one of the most important factors determining sudden cardiac death (SCD) risk. In a large unselected sample of HCM patients, we aimed to simulate what changes would occur in the calculated SCD risk according to the European HCM Risk-SCD calculator when MWT measured using echocardiography was changed to MWT measured using MRI. All consecutive patients with HCM who underwent cardiac MRI were included. MWT measured with echocardiography and MRI were compared, and 5-year SCD risk according to the HCM Risk-SCD calculator was computed using four different models. The final population included 673 patients [389 (57.8%) males, median age 50 years, interquartile range (36–60)]. The median MWT was lower measured by echocardiography than by MRI [20 (17–24) mm vs 21 (18–24) mm; *p* < 0.0001]. There was agreement between echocardiography and MRI in the measurement of maximal LV wall thickness in 96 patients (14.3%). The largest differences between echo and MRI were − 13 mm and + 9 mm. The differences in MWT by echocardiography and MRI translated to a maximal difference of 8.33% in the absolute 5-year risk of SCD**,** i.e., the echocardiography-based risk was 8.33% lower than the MRI-based estimates. Interestingly, 13.7% of patients would have been reclassified into different SCD risk categories if MRI had been used to measure MWT instead of echocardiography. In conclusion, although there was high general intermodality agreement between echocardiography and MRI in the MWT measurements, the differences in MWT translated to significant differences in the 5-year risk of SCD.

## Introduction

In patients with hypertrophic cardiomyopathy (HCM), left ventricular (LV) maximal wall thickness (MWT) is one of the most important factors determining sudden cardiac death (SCD) risk^1,2^. According to American guidelines, LV MWT equal to or greater than 30 mm is considered a major risk factor for SCD^2,3^. In the European guidelines on the diagnosis and management of HCM, LV MWT is a part of the HCM Risk-SCD calculator^1^. Although the diagnosis of HCM can be made using any imaging modality (i.e., echocardiography, magnetic resonance imaging—MRI, cardiac computed tomography) able to identify LV hypertrophy equal to or greater than 15 mm, the risk of SCD is calculated based on LV MWT as measured with the use of echocardiography^1^. It has been demonstrated that discrepancies exist in measurements of MWT between echocardiography and MRI^4–10^. Considering MRI-based short axis measurements of LV MWT as a reference standard, echocardiography leads to discrepant results as a consequence of either overestimation or underestimation of LV MWT. Furthermore, this intermodality disagreement has been shown to lead to divergences in SCD risk estimation^5^. However, those reports are either based on relatively small study samples, which means that the reference bias cannot be excluded and/or did not consider differences in SCD risk assessment caused by inconsistent measurements of LV MWT. No previous work has addressed the issue of changes in estimated SCD risk depending on the method of measurements of LV MWT (echocardiography-based vs. MRI-based) in a large cohort of HCM patients. Accordingly, in a large unselected sample of HCM patients, we aimed to simulate what changes would occur in the calculated risk of SCD according to the European calculator when LV MWT measured with the use of echocardiography was changed to LV MWT measured using MRI.

## Results

### Baseline characteristics

There were 785 patients with HCM analysed during the study period [the analysis spanned 10 years (March 2008–March 2018)].

**Figure 1:**
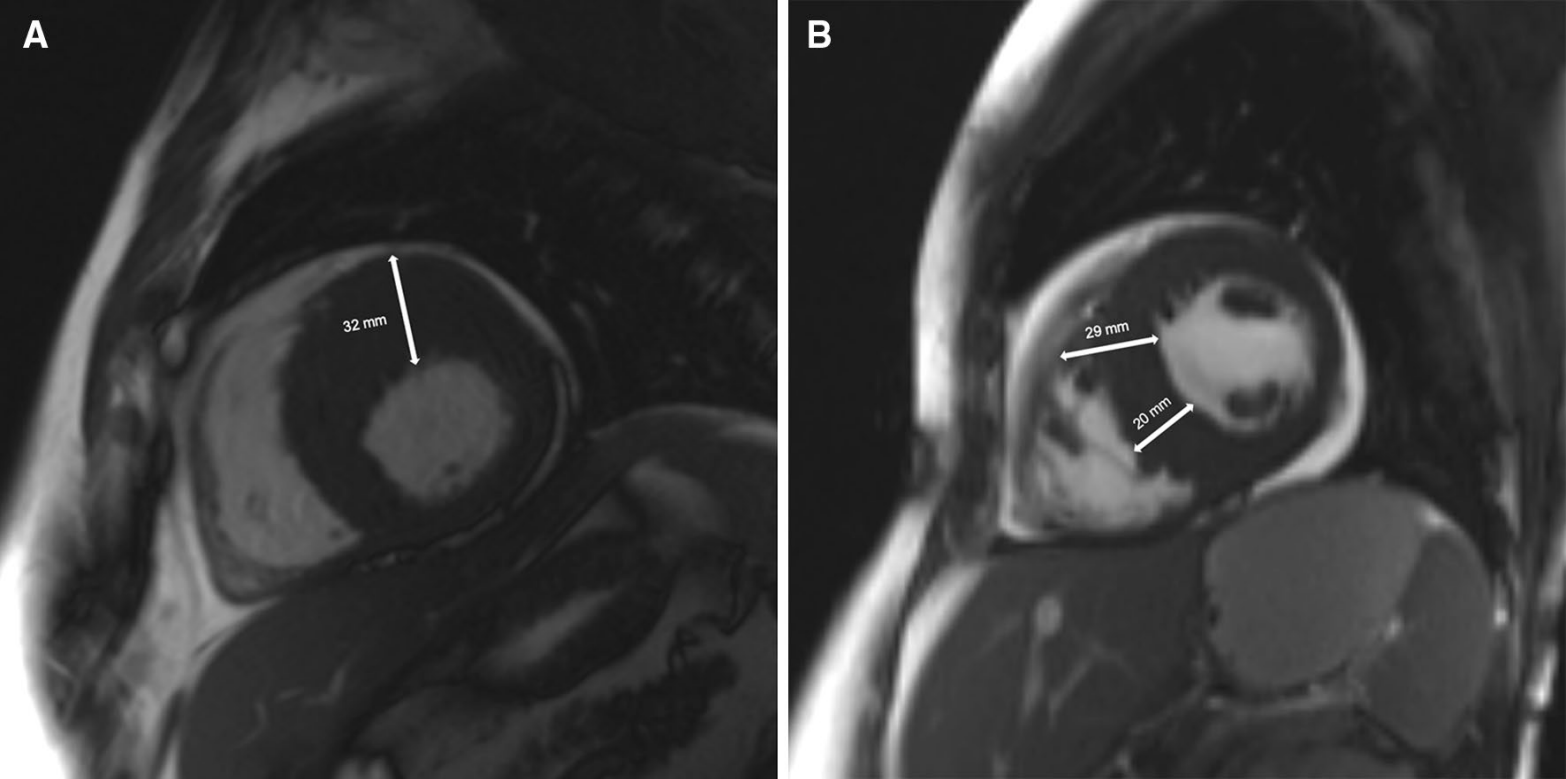
(**A**) Magnetic resonance image in short-axis slice demonstrating left ventricular hypertrophy with the maximal left ventricular wall thickness of 32 mm in basal anterior segment, which has been underestimated by 13 mm in transthoracic echocardiography. (**B**) Magnetic resonance image in short-axis slice demonstrating left ventricular hypertrophy with the maximal left ventricular wall thickness of 20 mm in mid infero-septal segment. The echocardiographic measurement of maximal left ventricular wall thickness (29 mm) was overestimated by inclusion of right ventricular trabeculae adjacent to the mid antero-septal segment.

Of these patients, we excluded those fulfilling the following exclusion criteria: history of septal reduction therapy, lack of echocardiographic dataset available, age < 16 years or > 81.4 years, LV MWT by echocardiography ≥ 35 mm or less than 10 mm, left atrium diameter either too small (< 28 mm) or too large (> 67 mm), and peak left ventricular outflow tract gradient higher than 154 mm Hg (Supplementary Fig. 1)^11^. The final population included 673 patients [389 (57.8%) males, median age 50 years, IQR = 36–60]. The median LV MWT measured by echocardiography was lower than that obtained by MRI [20 (17–24) mm vs 21 (18–24) mm; p < 0.0001]. There was a significant correlation between LV MWT measurements obtained by echocardiography and MRI (rho = 0.74, 95% CI = 0.70–0.77, p < 0.0001). Moreover, there was agreement between echocardiography and MRI in the measurement of maximal LV wall thickness in 96 patients (14.3%). In 228 patients (33.9%), echo measurements were higher than those obtained by MRI, and in 349 individuals (51.8%), echocardiography underestimated LV MWT compared to MRI. The highest difference between echo and MRI was − 13 mm [echo measurement (19 mm) was 13 mm lower than LV MWT by MRI (32 mm), Fig. 1A] and 9 mm [echo measurement (29 mm) was 9 mm higher than MWT by MRI (20 mm), Fig. 1B].

**Figure 2:**
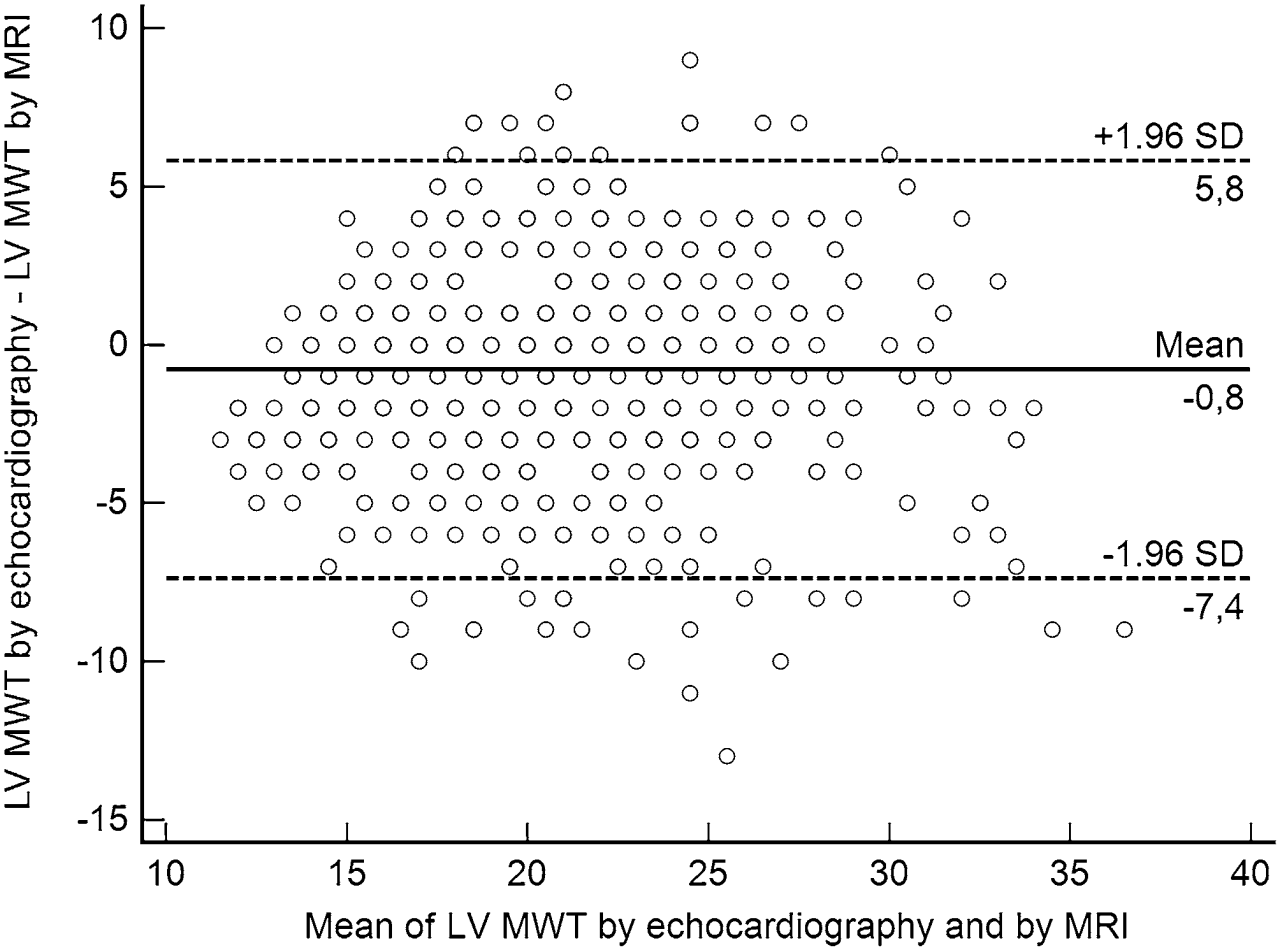
Bland–Altman plot demonstrating differences between left ventricular maximal wall thickness (LV MWT) by echocardiography and by MRI.

The median difference between echo and MRI was − 1 mm (i.e. echocardiography underestimated the MWT by 1 mm compared to MRI). The IQR was − 3–1 mm. A Bland–Altman plot demonstrating differences between LV MWT by echocardiography and MRI is presented in Fig. 2. The mean difference between LV MWT by echocardiography and by MRI was − 0.8 [95% confidence interwall (CI) =  − 1.0 to − 0.5].

### SCD risk according to Model 1 (best clinical scenario)

**Figure 3:**
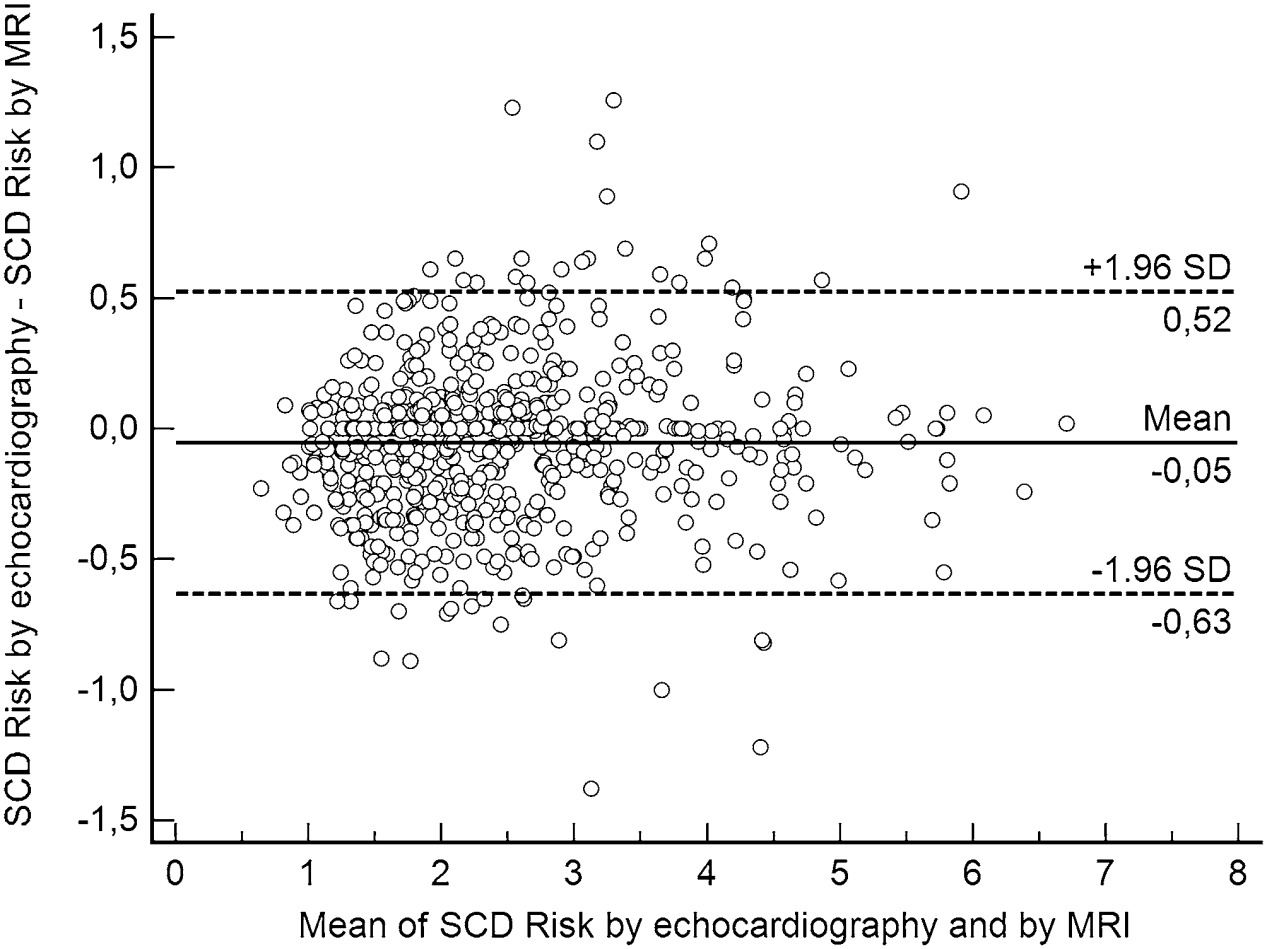
Bland–Altman plot demonstrating differences between echocardiography- and MRI-derived SCD risk estimates according to Model 1 (best clinical scenario).

In Model 1, the best clinical scenario (i.e., no clinical risk factors for SCD such as FHSCD, NSVT, or unexplained syncope), there was a median difference of − 0.01 in the estimated 5-year risk of SCD (IQR: − 0.22–0.08; range − 1.38–1.26), which means that in the extreme case, the echocardiography-based risk was 1.38% lower than the MRI-based risk estimates (Fig. 3). There was very good agreement in 5-year SCD risk categories (i.e., < 4%, 4–6%, ≥ 6%) between echocardiography and MRI (kappa = 0.88, 95% CI 0.82–0.94) (Table 1). In 658 patients (97.8%), both methods indicated the same risk category. However, in 9 of 613 patients (1.5%) in the echo-derived low-risk category, MRI indicated an intermediate risk of SCD (i.e., 4–6% over 5 years) (Table 1). In the echo-derived intermediate-risk category out of 56 patients, 5 patients (8.9%) were reclassified: 4 into the low-risk category and 1 into the high-risk category (Table 1). Finally, 1 of 4 patients (25%) with echo-derived high-risk category had intermediate risk according to LV MWT by MRI (Table 1).

**Table 1:** Comparison between echocardiography-derived and MRI-derived sudden cardiac death risk categories according to Model 1 (best clinical scenario, no clinical risk factors for SCD).

	Risk categories by echocardiography			
	< 4%	4–6%	≥ 6%	
**Risk categories by MRI**				
< 4%	**604**	4	0	608 (90.3%)
4–6%	9	**51**	1	61 (9.1%)
≥ 6%	0	1	**3**	4 (0.6%)
	613 (91.1%)	56 (8.3%)	4 (0.6%)	673

Bolded text indicates agreement between echo-derived and MRI-derived risk categories.

A Bland–Altman plot demonstrating differences between echocardiography- and MRI-derived SCD risk estimates according to Model 1 is presented in Fig. 3.

### SCD risk according to Model 2 (FHSCD scenario)

**Figure 4:**
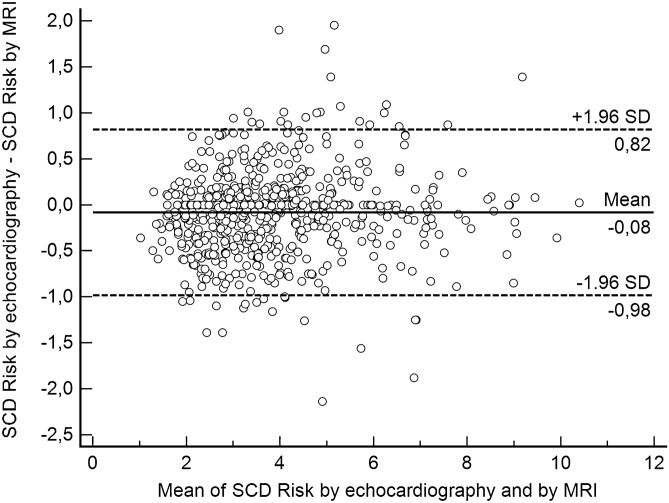
Bland–Altman plot demonstrating differences between echocardiography- and MRI-derived SCD risk estimates according to Model 2 (FHSCD scenario).

In Model 2 (FHSCD scenario), there was a median difference of − 0.01 in the estimated 5-year risk of SCD (IQR: − 0.35–0.13; range − 2.14–1.95*)*, i.e., the maximal echocardiography-based risk was 2.14% lower than the MRI-based risk estimates (Fig. 4). On the other hand, in another patient, the MRI-derived SCD risk was 1.95% lower than the echocardiography-derived SCD risk (Fig. 4). There was very good agreement in the 5-year SCD risk categories between echocardiography and MRI (kappa = 0.85, 95% CI 0.82–0.88) (Table 2). In 607 patients (90.2%), both methods indicated the same risk category (Table 2). However, 30/426 (7.0%) patients in the echo-derived low-risk category, 27/177 (15.3%) in the echo-derived intermediate-risk category, and 9 out of 70 (12.9%) in the echo-derived high-risk category were reclassified when SCD risk was estimated based on LV MWT by MRI (Table 2).

**Table 2:** Comparison between echocardiography-derived and MRI-derived sudden cardiac death risk categories according to Model 2 (FHSCD scenario).

	Risk categories by echocardiography			
	< 4%	4–6%	≥ 6%	
**Risk categories by MRI**				
< 4%	**396**	18	0	414 (61.5%)
4–6%	30	**150**	9	189 (28.1%)
≥ 6%	0	9	**61**	70 (10.4%)
	426 (63.3%)	177 (26.3%)	70 (10.4%)	673

Bolded text indicates agreement between echo-derived and MRI-derived risk categories.

A Bland–Altman plot demonstrating differences between echocardiography- and MRI-derived SCD risk estimates according to Model 2 is presented in Fig. 4.

### SCD risk according to Model 3 (FHSCD + NSVT scenario)

**Figure 5:**
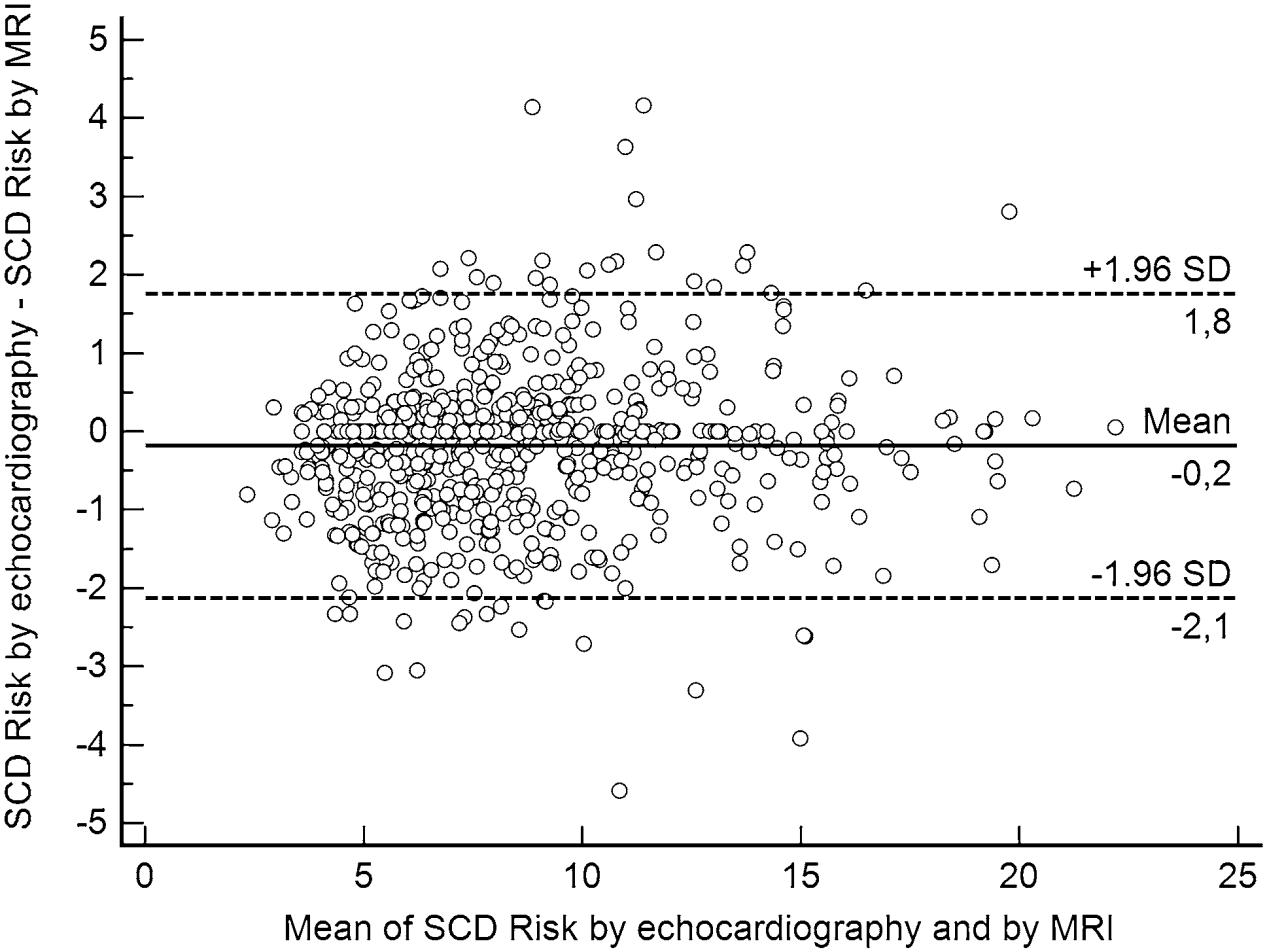
Bland–Altman plot demonstrating differences between echocardiography- and MRI-derived SCD risk estimates according to Model 3 (FHSCD + NSVT scenario).

In Model 3 (FHSCD + NSVT scenario), there was a median difference of − 0.02 in the estimated 5-year risk of SCD (IQR: − 0.74–0.29; range − 4.59–4.17), which means that in the extreme case, the echocardiography-derived risk was 4.59% lower than the MRI-derived risk estimates (Fig. 5). There was good agreement in the 5-year SCD risk categories between echocardiography and MRI (kappa = 0.69, 95% CI = 0.64–0.75) (Table 3). In 581 patients (86.3%), both methods indicated the same risk category. Out of 38 patients in the low-risk echo-based category, 18 (47.4%) were reclassified (mainly into the low-risk category) when MRI was used for measurements of LV MWT (Table 3). In the intermediate-risk echo-derived group, 48 out of 88 patients (54.5%) were reclassified (mainly into the high-risk group) when risk was estimated based on MRI-derived LV MWT. Finally, out of 499 patients in whom echocardiography indicated high risk, 23 (4.6%) had intermediate risk as indicated by MRI (Table 3).

**Table 3:** Comparison between echocardiography-derived and MRI-derived sudden cardiac death risk categories according to Model 3 (FHSCD + NSVT scenario).

	Risk categories by echocardiography			
	< 4%	4–6%	≥ 6%	
**Risk categories by MRI**				
< 4%	**17**	4	0	21 (3.1%)
4–6%	20	**88**	23	131 (19.5%)
≥ 6%	1	44	**476**	521 (77.4%)
	38 (5.6%)	136 (20.2%)	499 (74.2%)	673

Bolded text indicates agreement between echo-derived and MRI-derived risk categories.

Figure 5 demonstrates the Bland–Altman plot with differences between echocardiography- and MRI-derived SCD risk estimates according to Model 3.

### SCD risk according to Model 4 (worst clinical scenario)

**Figure 6:**
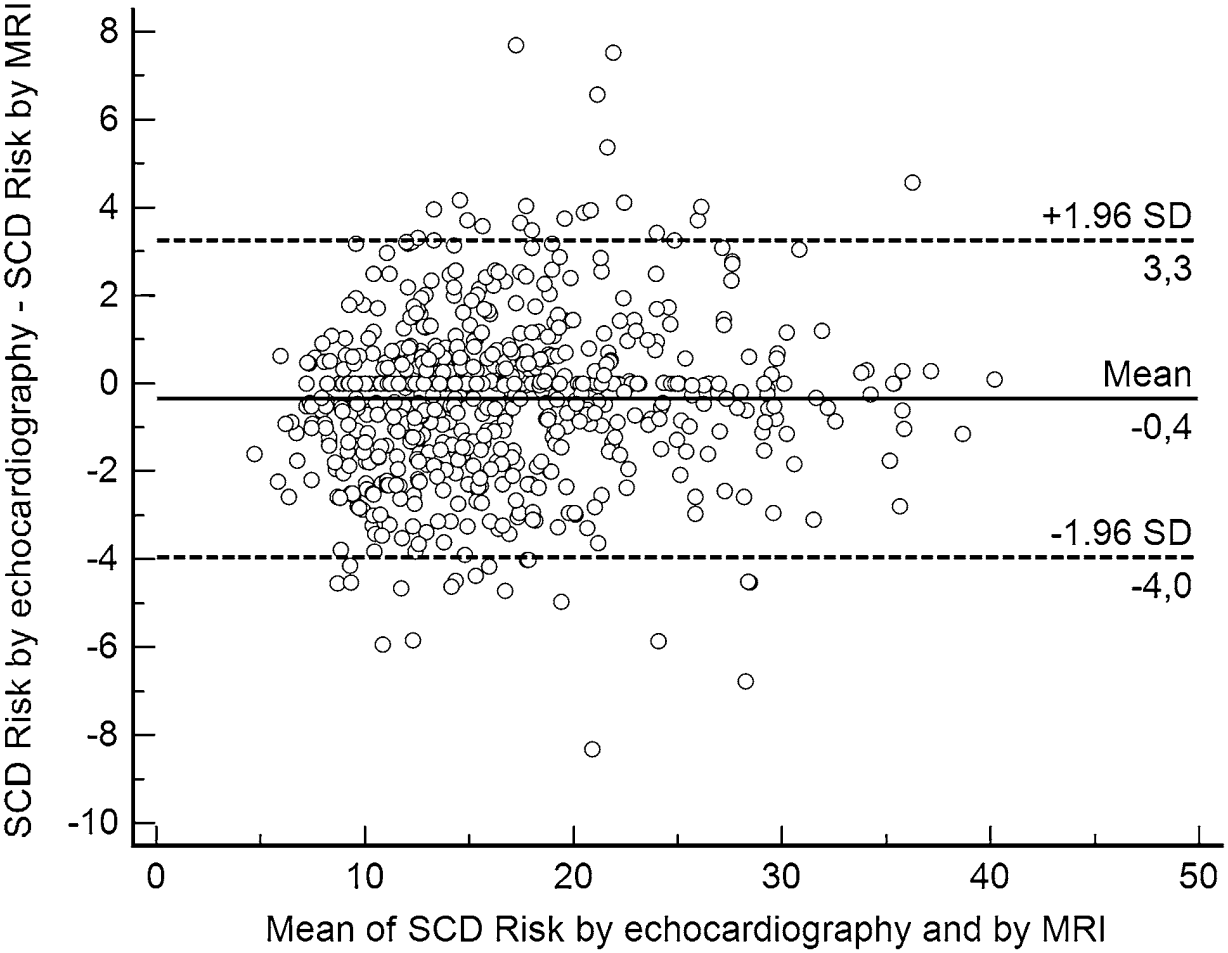
Bland–Altman plot demonstrating differences between echocardiography- and MRI-derived SCD risk estimates according to Model 4 (worst clinical scenario).

In Model 4, the worst clinical scenario (i.e., all clinical risk factors for SCD such as FHSCD, NSVT, and unexplained syncope), the median difference in the estimated 5-year risk of SCD was − 0.04 (IQR: − 1.40–0.55; range − 8.33–7.69), i.e., the echocardiography-based risk was 8.33% lower than the MRI-based estimates (Fig. 6). On the other hand, in another patient, MRI indicated a 7.69% lower SCD risk than echocardiography (Fig. 6). Although both methods indicated the same risk category in 667 out of 673 patients (99.1%), this result was driven only by agreement in high-risk groups (Table 4). There were too few patients in the low (< 4%) and intermediate (4–6%) risk categories to draw reliable conclusions about agreement between the two methods. In other words, the data show that there is good correspondence for the high-risk category (≥ 6%), but no conclusion can be made for the low- and intermediate-risk categories. Thus, one cannot say that overall, there was good correspondence. This was reflected by the low kappa value (0.25) and wide CI (95% CI = − 0.09–0.58).

**Table 4:** Comparison between echocardiography-derived and MRI-derived sudden cardiac death risk categories according to Model 4 (worst clinical scenario).

	Risk categories by echocardiography			
	< 4%	4–6%	≥ 6%	
**Risk categories by MRI**				
< 4%	**0**	0	0	0 (0.0%)
4–6%	1	**0**	1	2 (0.3%)
≥ 6%	0	4	**667**	671 (99.7%)
	1 (0.1%)	4 (0.6%)	668 (99.3%)	673

Bolded text indicates agreement between echo-derived and MRI-derived risk categories.

In Fig. 6, we demonstrate a Bland–Altman plot with differences between echocardiography- and MRI-derived SCD risk estimates according to Model 4.

## Discussion

The main findings of our study are as follows:

1. Although in general there was high intermodality agreement between echocardiography and MRI in the measurements of LV MWT (low median and mean difference between two methods), the maximal difference between the two modalities reached 13 mm.
2. The differences in LV MWT by echocardiography and MRI translated to a maximal difference of 8.33% in the absolute 5-year risk of SCD.
3. Overall, the estimated risk by echocardiography was lower than that observed when MRI was used to calculate the SCD risk.
4. The difference in the estimated risk of SCD increased with an increasing number of clinical risk factors for SCD (i.e., FHSCD, NSVT, and unexplained syncope).
5. Using the HCM Risk-SCD calculator, up to 14% of patients would have been reclassified into different SCD risk categories if, instead of echocardiography, MRI had been used for measurements of LV MWT.

The novel finding of our study is that the difference between echocardiography and MRI in the estimated risk of SCD increased with an increasing number of clinical risk factors for SCD. This underscores the importance of precise measurements of LV MWT, especially in high-risk (based on clinical assessment) patients. Urbano-Moral and colleagues recently showed that contrast-enhanced echocardiography renders LV MWT measurements that are closer to those obtained by MRI^7^. Thus, contrast echocardiography should be considered when the quality of echocardiographic images is suboptimal due to a poor acoustic window determined by body habitus. In particular, one should consider using contrast agent when performing transthoracic echocardiography when MRI cannot be performed due to contraindications or limited availability.

LV hypertrophy in some areas of the LV wall may be missed by echocardiography, and only MRI contributes to the diagnosis of LV hypertrophy (HCM) and its magnitude^8,9,12,13^. In our study, there was a maximal difference of 13 mm in terms of underestimation of echo measurement compared to LV MWT by MRI (echocardiography indicated MWT of 19 mm, while MRI showed 32 mm, Fig. 1A). The reason for this discrepancy was that echocardiography was unable to accurately visualize the area of maximal LV hypertrophy in the basal segment of the anterior wall, which is a known handicap of 2-dimensional echocardiography^12^. This area of the left ventricle is particularly clinically important since Maron et al. demonstrated that the basal anterior free wall and contiguous portion of the anterior ventricular septum represent the most common areas of left LV wall thickening in HCM^12^.

On the other hand, the extreme difference (9 mm) in terms of overestimation of echocardiographic measurement of LV MWT compared to MRI (29 mm vs 20 mm) was due to the inclusion of right ventricular trabeculae, which led to inaccurate echo measurement of left ventricular wall thickness (Fig. 1B). In HCM patients, right ventricular hypertrophy might be present in addition to LV hypertrophy; therefore, cardiac MRI should be used to differentiate hypertrophic RV trabeculae adjacent to the interventricular septum from a truly hypertrophic interventricular septum^13–17^.

Similar to previous studies, we showed that the mean difference in LV MWT measured by echocardiography and MRI is low^4,5,7,8^. However, considering each patient separately, the difference in measurements may be of clinical significance since it may lead to reclassification of the patient’s individual SCD risk category. As shown in our study, this was the case in up to 14% of patients burdened with the most prevalent clinical risk factors for SCD, namely, FHSCD and NSVT. This is in line with a study by Webb et al., in which 6 out of 50 (12%) patients studied were reclassified when cardiac MRI was used instead of echocardiography to assess LV MWT^5^. While HCM remains the main cause of SCD in young persons, the incidence of SCD in HCM patients is low. Thus, it is of particular importance to select patients not requiring cardioverter-defibrillator implantation for primary prevention of SCD and not to omit those in whom primary prevention should be implemented.

In HCM, a nonlinear relationship exists between the risk of SCD and maximum left ventricular wall thickness^1,11^. Thus, in a risk prediction model, a quadratic term for maximum left ventricular wall thickness is included^1,11^. This underscores the importance of correct measurement of LV MWT since any discrepancy results in higher than linear difference in 5-year SCD risk. On the other hand, it needs to be remembered that the estimated risk of SCD tended to decrease with extreme hypertrophy measured as LV MWT ≥ 35 mm^11^.

According to Maron et al., the prognostic potential of the HCM Risk-SCD calculator is controversial and may lead to misclassification (as having low risk scores) of the majority of patients with SCD or appropriate implantable cardioverter-defibrillator interventions^18^. Their study was based on a much larger population, namely, 1629 patients recruited from two high-volume HCM centres. It would be interesting to determine whether SCD risk calculation (with the use of the European calculator) using MRI instead of echocardiography for measuring LV MWT would result in risk estimates that were closer to the actual prognosis. As shown in our study, a significant mismatch exists between MRI-based and echocardiography-based SCD risks. This may translate into different accuracies of MRI and echocardiography in estimating the SCD risk in HCM patients. This notion may be supported by the observation that overall, the estimated risk by echocardiography was lower than that observed when MRI was used to calculate the SCD risk. One may speculate that patients misclassified as having low risk scores based on echocardiography would have higher risk estimates based on MRI that are closer to the actual SCD risk (verified on the basis of SCD episodes or appropriate implantable cardioverter-defibrillator interventions).

### Limitations

Owing to the retrospective character of the study, we were unable to provide detailed individual information on all factors included in the HCM Risk-SCD calculator (i.e., the presence of non-sustained ventricular tachycardia, unexplained syncope, or family history of SCD). Instead, we built four models ranging from low-risk patients (best clinical scenario meaning the absence of all three clinical risk factors for SCD) to high-risk individuals (worst clinical scenario assuming the presence of all the clinical risk factors for SCD).

Although we demonstrated divergences in estimated 5-year SCD risk between echocardiography and MRI, it remains unknown which imaging modality (echocardiography or MRI) predicts SCD risk better. So far, there have been no studies addressing this issue. Such a study would be of great clinical value. However, it would require a large multicentre longitudinal cohort study. O’Mahony and colleagues developed the HCM Risk-SCD calculator based on 3675 consecutive patients from six centres^11^. This number exceeds the capabilities of a single-centre study, such as our investigation. Although our hospital is a high-volume HCM centre, we included all consecutive HCM patients studied with the use of MRI during a 10-year period. This topic warrants further investigation and should be adequately addressed in future multicentre prospective studies. Our results strongly support the need for such investigations.

## Conclusions

In conclusion, LV MWT measurements by echocardiography and MRI are not interchangeable, and using them to estimate the SCD risk may lead to reclassification of the patient’s individual risk category in up to 14% of patients. The difference in the predicted 5-year SCD risk between those two imaging modalities is higher in patients burdened with several clinical risk factors for SCD.

## Materials and methods

### Study population

All consecutive patients with HCM who had undergone cardiac MRI in a 10-year time span (2008–2017) were included. Patients examined in 2008–2014 were analysed retrospectively, and those examined in 2015–2017 were included prospectively. The study was approved by the ethics committee of the National Institute of Cardiology, Warsaw, Poland, (Reference number: 1656), and all patients included prospectively provided written informed consent. For patients included retrospectively, written informed consent was waived by the ethics committee of the National Institute of Cardiology, Warsaw, Poland (Reference number: 1656). The study was performed in accordance with the Declaration of Helsinki. We excluded patients younger than 16 years, those with HCM associated with metabolic diseases and syndromes, and individuals with a history of any septal reduction therapy (i.e., alcohol septal ablation or surgical myectomy)^1^. Additionally, we excluded all patients with advanced age, left atrial diameter, peak LV outflow tract gradient, or LV MWT exceeding the range in a cohort of patients included in the study from which the HCM Risk-SCD calculator was derived^11^.

All patients underwent standard-of-care transthoracic echocardiography with commercially available systems. LV MWT was recorded as the maximal wall thickness registered in short axis view.

All cardiac MRI studies were performed using a 1.5 T system (Avanto, Avanto^fit^, Siemens Healthineers). The standard protocol included a stack of cine (balanced steady state free precession) images covering both ventricles from the base to the apex and three long-axis cine images (2-, 3-, and 4-chamber view). The LV MWT was measured as the largest distance between the epicardium and endocardium in short-axis slices.

### Ethics approval

Patients who were included prospectively (recruited from 2015) provided written informed consent, and approval was granted by the local ethics committee for the retrospective analyses (Reference number: 1656).

### Calculation of sudden cardiac death risk

For calculation of the difference between SCD risk at 5 years using echocardiography-derived vs. MR-derived LV MWT, the following formula was used^1,11^:

$$ {\text{Probability}}_{{{\text{SCD}}\;{\text{at}}\;5\;{\text{years}}}} = 1{-}0.998\exp^{{({\text{Prognostic}}\;{\text{index}})}} $$

where Prognostic index = [0.15939858 × maximal wall thickness (mm)] − [0.00294271 × maximal wall thickness^2^ (mm^2^)] + [0.0259082 × left atrial diameter (mm)] + [0.00446131 × maximal (rest/Valsalva) left ventricular outflow tract gradient (mm Hg)] + [0.4583082 × family history of SCD] + [0.82639195 × NSVT] + [0.71650361 × unexplained syncope] − [0.01799934 × age at clinical evaluation (years)], where NSVT means non-sustained ventricular tachycardia on ECG Holter monitoring.

The difference between echocardiography-based and MRI-based risk was calculated as follows:

$$ \begin{aligned} {\text{Probability}}_{{{\text{difference}}}} & = {\text{Probability}}_{{{\text{echo}}}} {-}{\text{Probability}}_{{{\text{MRI}}}} \\ & = \left( {1{-}0.998\exp^{{({\text{Prognostic}}\;{\text{index}}\;{\text{by}}\;{\text{echo}})}} } \right){-}\left( {1{-}0.998\exp^{{({\text{Prognostic}}\;{\text{index}}\;{\text{by}}\;{\text{MRI}})}} } \right) \\ \end{aligned} $$

where Prognostic index by echo = Prognostic index = [0.15939858 × maximal wall thickness by echo (mm)] − [0.00294271 × maximal wall thickness by echo^2^ (mm^2^)] + [0.0259082 × left atrial diameter (mm)] + [0.00446131 × maximal (rest/Valsalva) left ventricular outflow tract gradient (mm Hg)] + [0.4583082 × family history of SCD] + [0.82639195 × NSVT] + [0.71650361 × unexplained syncope] − [0.01799934 × age at clinical evaluation (years)], and Prognostic index by MRI = Prognostic index = [0.15939858 × maximal wall thickness by MRI (mm)] − [0.00294271 × maximal wall thickness by MRI^2^ (mm^2^)] + [0.0259082 × left atrial diameter (mm)] + [0.00446131 × maximal (rest/Valsalva) left ventricular outflow tract gradient (mm Hg)] + [0.4583082 × family history of SCD] + [0.82639195 × NSVT] + [0.71650361 × unexplained syncope] − [0.01799934 × age at clinical evaluation (years)].

Owing to the partially retrospective character of the study, we were unable to provide detailed clinical characteristics of the study group in terms of the presence of clinical risk factors for SCD included in the HCM Risk-SCD calculator [i.e.: family history of SCD (FHSCD), NSVT, and unexplained syncope]. Instead, we have built 4 models according to the presence of those factors:

- Model 1 (best clinical scenario) assuming that none of the patients had any of the clinical risk factors for SCD,
- Model 2 assuming that all patients had FHSCD as a sole clinical risk factor for SCD; FHSCD was the most common clinical risk factor for SCD in the study by O’Mahony et al.^11^,
- Model 3 assuming that all patients had FHSCD and NSVT as clinical risk factors for SCD; NSVT was the second most prevalent clinical risk factor for SCD in the study by O’Mahony et al.^11^,
- Model 4 (worst clinical scenario) assuming that all patients had all three clinical risk factors for SCD (unexplained syncope in addition to FHSCD and NSVT); unexplained syncope was the least common clinical risk factor for SCD in the study by O’Mahony et al.^11^.

After calculating the estimated 5-year SCD risk according to each model for each patient, we categorized patients into the following risk groups according to the current guidelines^1^:

- low-risk group (i.e., estimated risk < 4%)
- intermediate-risk group (i.e., estimated risk 4–6%)
- high-risk group (i.e., estimated risk ≥ 6%).

### Statistical analysis

Continuous data were tested for a normal distribution using the Kolmogorov–Smirnov test and are presented as the median with interquartile range (IQR) as appropriate for data without a normal distribution. The comparison between LV MWT by echocardiography and by MRI was performed using the Wilcoxon rank sum test, as appropriate for non-normally distributed variables. To test the agreement between SCD risk categories based on echo- or MRI-derived LV MWT measurements, we used weighted Cohen’s kappa. To demonstrate mean differences between LV MWT measured by echocardiography versus MRI, as well as to demonstrate mean differences in the estimated 5-year SCD risk in various models (Models 1–4, please see above), we used Bland–Altman plots. The Spearman coefficient was used to assess the correlation between the two methods of assessment of LV MWT.

A two-sided p-value of 0.05 or less was considered statistically significant. Statistical analysis was performed with the use of MedCalc software, version 19.4.1 (MedCalc Software Ltd, Ostend, Belgium).

## Supplementary Information

Supplementary Information
