# Supplementary material for: Sudden cardiac death risk in hypertrophic cardiomyopathy: comparison between echocardiography and magnetic resonance imaging
Source: Sci Rep. 2021 Mar 30;11:7146. doi: 10.1038/s41598-021-86532-4 (PMC8009882; doi:10.1038/s41598-021-86532-4)
Supplement: Supplementary file 1 — Supplementary Information [file 41598_2021_86532_MOESM1_ESM.pdf]

Supplementary Figure 1

Sudden cardiac death risk in hypertrophic cardiomyopathy – comparison between echocardiography and magnetic resonance imaging

Mateusz Śpiewak[1], Mariusz Kłopotowski[2], Ewa Kowalik[3], Agata Kubik[1], Natalia Ojrzyńska-Witek[4], Joanna Petryka-Mazurkiewicz[5], Ewa Michalak[6], Łukasz Mazurkiewicz[4], Monika Gawor[4], Katarzyna Kożuch[3], Barbara Miłosz-Wieczorek[1], Jacek Grzybowski[4], Zofia Bilińska[6], Adam Witkowski[2], Anna Klisiewicz[3], Magdalena Marczak[1]

[1] Magnetic Resonance Unit, Department of Radiology, Institute of Cardiology, Warsaw, Poland
[2] Department of Cardiology and Interventional Angiology, Institute of Cardiology, Warsaw, Poland
[3] Department of Congenital Heart Diseases, Institute of Cardiology, Warsaw, Poland
[4] Department of Cardiomyopathies, Institute of Cardiology, Warsaw, Poland
[5] Department of Coronary Artery Disease and Structural Heart Diseases, Institute of Cardiology, Warsaw, Poland
[6] Unit for Screening Studies in Inherited Cardiovascular Diseases, Institute of Cardiology, Warsaw, Poland

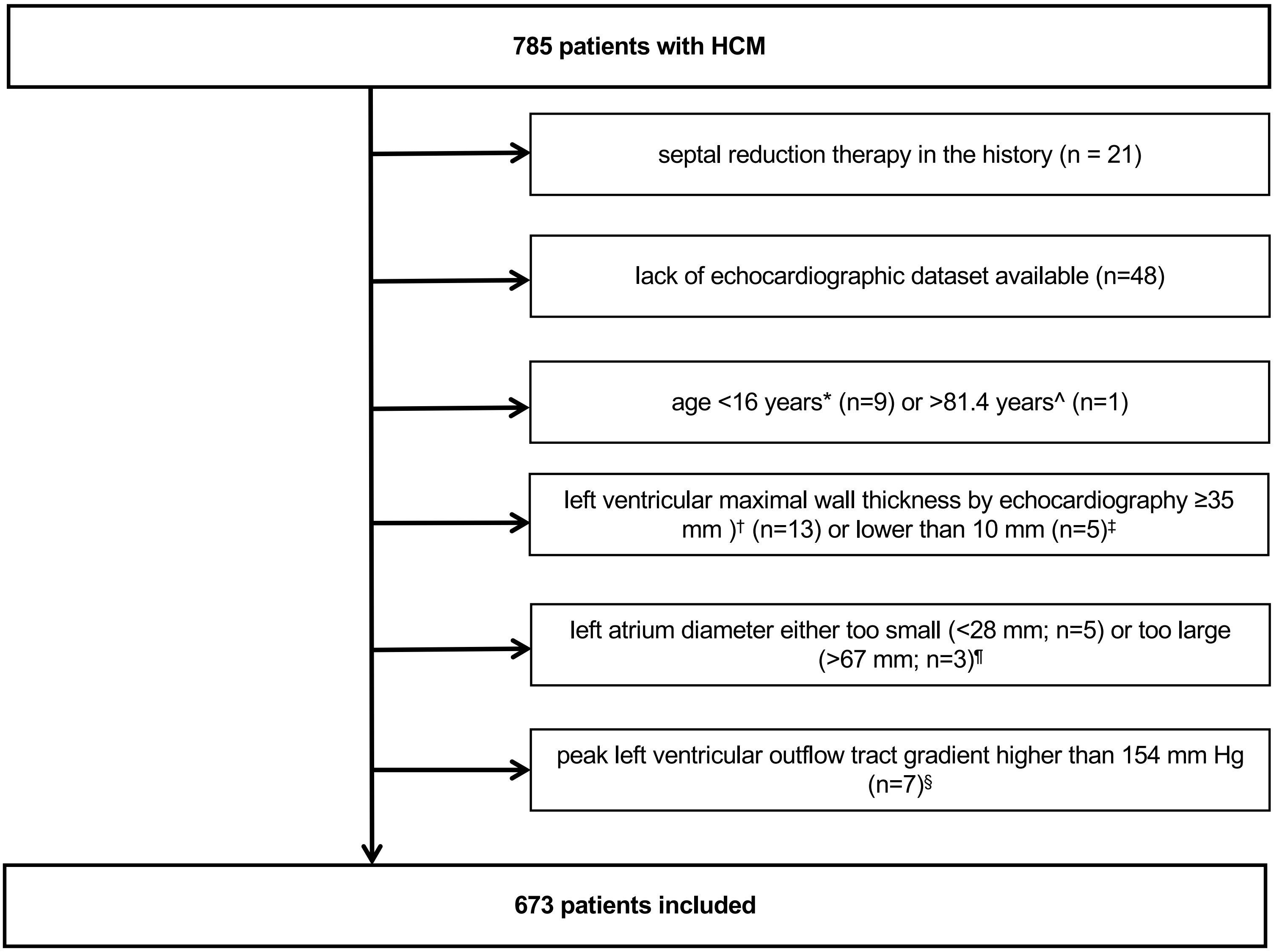

* HCM Risk-SCD calculator is not recommended for use with patients younger than 16 years old
^ In the study by O'Mahony et al. [11], the upper limit of the age range was 81.4 years
† HCM Risk-SCD calculator should be used cautiously in patients with left ventricular maximal wall thickness ≥35 mm
‡ In the study by O'Mahony et al. [11] the lower limit of the range was 10 mm
¶ In the study by O'Mahony et al. [11] the lower and upper limits of the range were 28 mm and 67 mm, respectively
§ In the study by O'Mahony et al. [11] the upper limit of the range was 154 mm Hg
